# Impact of COVID-19 on Psychiatric Patients: The Role of Vaccination, Comorbidities, and Biomarkers in Clinical Outcomes

**DOI:** 10.3390/jcm13195950

**Published:** 2024-10-07

**Authors:** Konstantinos Argyropoulos, Aikaterini-Aggeliki Argyropoulou-Grizanou, Eleni Jelastopulu

**Affiliations:** 1 Department of Public Health, School of Medicine, University of Patras, 26504 Patras, Greece; konargyropoulos@yahoo.gr; 2 Department of Social Sciences, Hellenic Open University, 26335 Patras, Greece; std512255@ac.eap.gr

**Keywords:** biomarkers, COVID-19, CRP, ferritin, psychiatric patients

## Abstract

**Background/Objectives**: The COVID-19 pandemic has posed significant challenges, particularly for individuals residing in psychiatric facilities. This study aims to investigate the impact of COVID-19 on psychiatric patients, focusing on factors such as their vaccination status, comorbidities, medication regimens, and biomarkers like C-reactive protein (CRP) and ferritin. **Methods**: This retrospective study analyzed 100 patients with confirmed SARS-CoV-2 infections admitted to the private psychiatric clinic “Asclepius of Upper Volos” from March 2020 to March 2023. The data collected included sociodemographic characteristics, vaccination status, symptom severity, medication regimens, and levels of CRP and ferritin. Statistical analyses using IBM SPSS Statistics version 29 included Pearson’s chi-square tests, Student’s t-tests, and a survival time analysis via the log-rank test to assess associations between clinical characteristics and outcomes. **Results**: Among the participants, 64% were female and 74% received two doses of the COVID-19 vaccine. The majority experienced mild symptoms, with a survival rate of 74%. Statistically significant findings include a higher survival rate among vaccinated individuals (98.6%) versus unvaccinated individuals (1.4%, *p* < 0.001). Comorbidities like chronic obstructive pulmonary disease (COPD), coronary artery disease, and renal failure were associated with severe symptoms and higher mortality rates. Higher ferritin levels were significantly associated with poorer outcomes, with survivors having a mean ferritin level of 246.2 (SD = 150.3) compared to the 416.9 (SD = 215.9) seen in non-survivors (*p* < 0.001). Similarly, mean CRP levels were lower in survivors (1.58, SD = 1.96) than in non-survivors (3.46, SD = 2.92), with a *p*-value of 0.002. **Conclusions**: The findings underscore the importance of tailored health protocols and continued support for this vulnerable population. Enhanced strategies for managing comorbidities and utilizing biomarkers can aid in better predicting and improving psychiatric patient outcomes.

## 1. Introduction

The COVID-19 pandemic, caused by the SARS-CoV-2 virus, has profoundly impacted various populations worldwide. Officially declared a pandemic by the World Health Organization (WHO) on 11 March 2020, COVID-19 has resulted in more than 590 million confirmed cases and 6.4 million deaths globally as of August 2022 [1]. The virus spreads through respiratory droplets, which can be inhaled or contaminate surfaces, leading to a wide spectrum of severity. While some individuals remain asymptomatic or experience mild symptoms like a cough and fever, others develop severe respiratory infections, necessitating advanced medical care [2].

Among the most vulnerable groups affected by the pandemic are individuals residing in psychiatric facilities. These environments, characterized by close living quarters and limited space, present a high-risk setting for SARS-CoV-2 transmission. The conditions in many psychiatric facilities often fall short of the health standards needed to effectively manage an epidemic, making containment and isolation efforts particularly challenging. Enhancing health protocols and support within these settings is essential to protect this vulnerable population and manage the pandemic more effectively in this context [3,4].

Psychiatric patients have faced unique challenges during the COVID-19 pandemic, which have been exacerbated by the conditions imposed by the virus’s spread. One key factor is the high prevalence of comorbidities among this population. Psychiatric patients often exhibit high rates of conditions like obesity and metabolic syndrome, which exacerbated their health risks during the pandemic. Additionally, chronic obstructive pulmonary disease (COPD), often associated with the higher smoking rates in psychiatric facilities, presents further challenges in managing patient health [5].

Medication management poses another significant challenge. Many psychiatric patients are on medications that can influence their immune system or their response to infections. For example, some antipsychotic drugs used to treat conditions like schizophrenia or bipolar disorder may impact the body’s ability to react to infections, potentially complicating COVID-19 treatment and prevention efforts [6,7]. This situation necessitates careful monitoring and the potential adjustment of medication regimens to mitigate adverse effects on patients’ overall health during the pandemic.

Another critical issue is the difficulty many psychiatric patients face in adhering to COVID-19 preventive measures. Severe mental illnesses, such as depression and schizophrenia, can significantly impair patients’ ability to follow daily precautions, such as mask-wearing, hand hygiene, and social distancing. The cognitive and behavioral challenges associated with these conditions make it harder for patients to understand and consistently practice preventive behaviors, increasing their risk of infection and transmission within psychiatric facilities [8,9].

Vaccination remains the most effective and, in many cases, the only reliable treatment for preventing severe COVID-19 outcomes in psychiatric patients [10].

Psychiatric patients may have mixed attitudes toward vaccination. While some individuals are open to vaccination, others may harbor significant concerns about side effects, distrust the medical system, feel misunderstood by healthcare providers, or have a greater belief in conspiracy theories. A systematic review found no studies examining the attitudes of individuals with mental illness regarding vaccination against COVID-19 [11]. Ensuring vaccine access for individuals with severe mental illness involves addressing structural and individual barriers such as community outreach, integration with mental health services, tailored education, and targeted vaccination programs [11]. Clinicians face a delicate ethical balance when a mentally ill person refuses vaccination. On the one hand, respecting a patient’s autonomy and bodily integrity is a core principle of medical ethics, and forced vaccination may violate this autonomy. This challenge is heightened if the individual’s ability to make informed decisions is impaired by their mental illness [12].

Vaccinations are generally safe, but neuropsychiatric side effects, though rare, can occur. Studies have reported transient neuropsychiatric symptoms such as cognitive impairment, mood changes, sleep disturbances, increased psychomotor activity, and even psychosis, particularly in individuals with pre-existing psychiatric disorders, following vaccination [11]. There is limited evidence of direct interactions between vaccines and psychotropic medications. However, the immunomodulatory effects of certain psychiatric drugs, such as clozapine or lithium, could theoretically influence vaccine efficacy [13,14]. Severe mental illness can affect vaccine responses in multiple ways, including through immune dysfunction due to chronic stress, poor nutrition, or the effects of medications. Psychiatric patients in institutions have reported weaker immune responses after vaccination compared to the general population [15].

Among the biomarkers commonly studied in the context of COVID-19, C-reactive protein (CRP) and ferritin have emerged as important indicators of disease severity and clinical outcomes in psychiatric patients. Both of these inflammatory markers play significant roles in the body’s response to infection and are associated with worse clinical outcomes when elevated. Elevated CRP levels have been consistently associated with severe COVID-19 outcomes, including higher rates of hospitalization, progression to acute respiratory distress syndrome (ARDS), and mortality [16]. In psychiatric patients, the prognostic role of CRP may be particularly important due to the frequent presence of comorbidities such as metabolic syndrome, cardiovascular disease, and obesity, all of which independently contribute to higher CRP levels. Studies have shown that patients with severe mental illnesses often have elevated baseline CRP levels, even without infection, due to the chronic low-grade inflammation associated with psychiatric disorders such as schizophrenia and major depressive disorder [17]. This pre-existing inflammatory state can compound the effects of an acute infection like COVID-19, leading to more severe clinical outcomes. Moreover, psychiatric medications, particularly antipsychotics, can modulate inflammatory responses, further influencing CRP levels during infection [18]. This suggests that monitoring CRP levels in this population can provide valuable insights into the severity of the disease and may help guide therapeutic decisions, such as the early initiation of anti-inflammatory treatments.

Ferritin, another key biomarker of inflammation, is a protein responsible for iron storage and is released during infection as part of the body’s immune response. Elevated serum ferritin levels are commonly observed in patients with severe COVID-19 and have been associated with hyperinflammatory states such as a cytokine storm, which can lead to multiorgan failure and death [19]. In psychiatric patients, ferritin levels may be elevated due to chronic inflammation or iron dysregulation, particularly in those with conditions such as metabolic syndrome or liver disease, which are prevalent in this population. Elevated ferritin levels in psychiatric patients with COVID-19 have been shown to correlate with worse clinical outcomes, including higher rates of severe disease, ARDS, and mortality [20]. Furthermore, psychiatric patients with elevated ferritin levels may be more susceptible to the hyperinflammatory responses seen in severe COVID-19, increasing their risk of complications. Ferritin has also been identified as a marker of disease severity in other infectious diseases, but its role in COVID-19 has been particularly prominent due to its strong association with the cytokine storm syndrome [21].

The COVID-19 pandemic has highlighted and exacerbated the vulnerabilities of psychiatric patients, particularly those residing in closed facilities. The unique challenges posed by this population, including high rates of comorbidities, medication-related complications, and difficulties adhering to preventive measures, underscore the need for targeted strategies to protect and support these individuals [22]. Enhanced health protocols, personalized care plans, and comprehensive support systems are essential to effectively manage the pandemic’s impact on psychiatric patients and ensure their well-being [23].

The purpose of the present study is to examine the impact of COVID-19 on psychiatric patients and to identify various risk factors, medication effects, and health outcomes by analyzing data on patients from a private psychiatric clinic in Greece. Key factors such as age, BMI, comorbidities, medication regimens, vaccination status, and biomarker levels of C-reactive protein (CRP) and ferritin are explored to understand how these variables influence the severity of the disease and patient outcomes.

## 2. Materials and Methods

The present study examines the impact of COVID-19 on 100 psychiatric patients, focusing on various risk factors, medication effects, and outcomes. The data were collected from the private psychiatric clinic “Asclepius of Upper Volos,” a facility with a capacity of 50 beds, located in Magnesia, central Greece.

Since the pandemic’s onset, psychiatric facilities have faced significant challenges due to their enclosed environments and the heightened vulnerability of their residents.

This retrospective study analyzes the total number of patients infected with SARS-CoV-2 between March 2020 and March 2023, examining factors such as age, BMI, comorbidities, medication regimens, vaccination status, and levels of C-reactive protein (CRP) and ferritin.

The inclusion criteria were a confirmed rapid test for SARS-CoV-2 infection, admission to the psychiatric facility, and a documented psychiatric diagnosis either before or during the patient’s stay at the clinic. Additionally, comprehensive medical records, including patients’ vaccination status (number of doses), drugs, comorbidities, and biomarker data (such as CRP and ferritin levels), were necessary for their inclusion.

Patients were excluded if they had incomplete medical records, did not test positive for COVID-19, or if their infection was not recorded in the national case registry. Individuals under the age of 18, as well as those transferred to other healthcare facilities before completing their COVID-19 treatment at the psychiatric clinic (due to a lack of follow-up data), were also excluded.

All patients who met the above-mentioned criteria during this period were included in the study (n = 100). Medical records were collected, with consent obtained from patients or guardians when the patients were incapacitated by the illness, ensuring confidentiality throughout the study. The study was conducted in accordance with the Declaration of Helsinki and approved by the Institutional Review Board of the private psychiatric clinic “Asclepius of Upper Volos”.

The collected information includes sociodemographic characteristics such as gender, place of residence (urban/rural), and marital status. Additionally, the year of admission to the clinic and SARS-CoV-2 vaccination status were recorded. The severity of the disease and patient outcomes were also documented.

For the statistical analysis, IBM SPSS Statistics version 29 (IBM, Chicago, IL, USA) was utilized. Continuous variables were summarized using the mean and standard deviation, providing a clear understanding of the central tendency and dispersion of the data. Categorical variables were described using absolute and relative frequencies to highlight the distribution and proportion of each category.

To explore the relationships between variables, Pearson’s chi-square test was employed for examining associations between two categorical variables. For comparing a continuous variable between two groups, Student’s t-test was applied, allowing us to determine if there were significant differences in their means.

Additionally, a survival time analysis based on patients’ clinical characteristics was performed using the log-rank test. This method provided insights into how various factors influenced patient outcomes over time. A significance level of 0.05 (5%) was set for all statistical tests, indicating that the findings were considered statistically significant if their *p*-value was less than 0.05.

## 3. Results

Table 1 summarizes the demographic characteristics of the study population. Of the sample, 64.0% were female, 74.0% of patients received two doses of the SARS-CoV-2 vaccine, and 50.0% experienced mild symptoms during the illness caused by the virus.

The most common comorbidity among participants was arterial hypertension, occurring in 50.0% of the cases, followed by arrhythmias, at 27.0%, and anemia, at 17.0%. Other frequently observed comorbidities included diabetes mellitus (15.0%), epilepsy (13.0%), and dyslipidemia (13.0%). The treatment that most of the patients received was antipsychotic medication, administered in 11.0% of cases, followed by antidepressants in 10.0% and antihypertensive therapy in 9.0%. Other common treatments included antithrombotic drugs (8.0%), antiepileptic medications (7.0%), and anti-dementia drugs (7.0%).

The statistical analysis results show no statistically significant difference in vaccination rates between male and female participants, with most individuals in both groups having received at least one dose. Furthermore, there was no significant difference in vaccination rates based on birthplace, the mean age between vaccinated and unvaccinated participants, or vaccination status in relation to Body Mass Index (BMI).

Table 2 shows that there is a statistically significant difference in symptom severity depending on vaccination status. Specifically, 97.4% of participants who were asymptomatic or had mild or moderate symptoms were vaccinated with at least one dose, while only 72.7% of participants with severe or critical symptoms were vaccinated with at least one dose.

According to Table 3, there is a statistically significant difference in outcomes based on vaccination status. Specifically, the survival rate among unvaccinated individuals who contracted SARS-CoV-2 was significantly lower (1.4%) compared to those who were vaccinated with at least one dose (98.6%).

Table 4 shows that there is a statistically significant association between the severity of symptoms and certain comorbidities. Specifically, the percentage of patients with chronic obstructive pulmonary disease (COPD) who experienced severe or critical symptoms was significantly higher (75.0%) compared to those without COPD. Similarly, patients with severe or critical symptoms were more frequent among those with coronary artery disease (80.0%) compared to those without (18.9%). The same pattern is observed for patients with renal failure and dyslipidemia, with these conditions being associated with a higher likelihood of severe or critical symptoms.

As expected, there is a statistically significant relationship between the severity of symptoms and the outcome experienced. Specifically, 84.6% of patients who developed severe or critical symptoms due to SARS-CoV-2 did not survive, in contrast to those who had mild or moderate symptoms or were asymptomatic, who all survived (100.0%) (Table 5).

Table 6 shows that there is a statistically significant relationship between the outcome experienced and certain comorbidities. For example, the survival rate among patients without anemia was significantly higher (79.5%) compared to those with anemia (47.1%). Moreover, no patients with renal failure survived, while 76.3% of those without renal failure did survive. Additionally, 76.8% of patients without coronary artery disease survived, compared to the 20.0% who survived with the disease.

According to Table 7, there is a statistically significant relationship between the outcome and antihypertensive treatment. Specifically, 82.1% of patients who did not receive antihypertensive treatment survived after contracting SARS-CoV-2, compared to only 63.6% of those who were on antihypertensive therapy. This likely reflects the link between SARS-CoV-2 infection and cardiovascular diseases.

According to Table 8, there is a statistically significant difference in mean ferritin levels between those who survived COVID-19 and those who did not. Specifically, the mean ferritin level in survivors was 246.2 units (SD: 150.3), compared to the 416.9 units (SD: 215.9) seen in non-survivors. Moreover, there is a statistically significant difference in mean CRP levels between those who survived COVID-19 and those who did not. Specifically, the mean CRP level in survivors was 1.58 units (SD: 1.96), compared to the 3.46 units (SD: 2.92) seen in non-survivors. No statistically significant difference was found in terms of mean BMI based on the patients’ outcomes.

Table 9 shows that a statistically significant difference in median survival times was found based on vaccination status. Specifically, those who were vaccinated with at least one dose had a median survival time of 35 days (95% CI: 7.9–28.1 days), while unvaccinated participants had a median survival time of 18.8 days (95% CI: 16.0–54.0 days). Additionally, the median survival time for asymptomatic patients or those with mild or moderate symptoms was significantly higher (50.0 days, 95% CI: 8.4–91.6 days) compared to those with severe or critical symptoms (18.0 days, 95% CI: 11.1–24.9 days). Regarding treatments, patients on antidepressants had a significantly higher median survival time compared to those not on such a treatment. As for comorbidities, patients with heart failure or coronary artery disease had significantly lower median survival times. Specifically, the median survival time for participants with heart failure was 18.0 days (95% CI: 11.9–24.1), compared to 28.0 days (95% CI: 20.6–35.5) for those without heart failure. Similarly, the median survival time for participants with coronary artery disease was 12.0 days (95% CI: 3.4–20.6), compared to 28.0 days (95% CI: 13.4–42.6) for those without the disease. Finally, both men and women with abnormal ferritin levels had lower median survival times.

## 4. Discussion

The results of our research largely align with findings from our literature review on COVID-19 cases among individuals with mental illnesses. Specifically, we observed that the severity of symptoms varied significantly between vaccinated and unvaccinated individuals. Unvaccinated patients exhibited more severe symptoms, even in cases where their vaccination included only a single dose. Vaccination remains the most effective and reliable method for preventing and controlling virus transmission. Moreover, it acts as a preventive measure, ensuring that symptoms are mild and clinically manageable in the event of an infection. It also reduces mortality risks, even in cases of comorbidities or chronic conditions. As vaccination efforts intensified, numerous studies examined vaccines’ effectiveness and safety and impacts on healthcare facilities, mortality, and disease progression. Studies have also explored the correlation between various factors—such as age, gender, polypharmacy, and comorbidities—and disease progression and vaccine efficacy [24].

In one study, the authors indicated that vaccination was slightly less effective against Delta variant-related deaths than other variants. However, studies following the initiation of the vaccination program showed significantly higher infection, hospitalization, and mortality rates among those not fully vaccinated compared to fully vaccinated individuals [24,25]. Similarly, in our study, the percentage of fully vaccinated patients who developed a critical illness was significantly lower, and they had a shorter average hospital stay.

Regarding gender, our research did not reveal any significant differences in disease severity, symptoms, or mortality rates between men and women. These results are consistent with other studies that have found age, rather than gender, to be the primary factor influencing disease outcomes. Age is thus considered a risk factor, with higher mortality in older adults due to factors such as weakened immune systems, comorbidities, polypharmacy, and more. Some research also suggests that men may experience worse clinical outcomes than women. A systematic review and meta-analysis found that post-COVID-19 infection symptoms were more frequent in men than women. Additionally, the article noted an association between the male gender and severe symptoms and mortality [26].

A statistically significant relationship was found between outcomes and certain comorbidities in the present study. One study aimed to identify the comorbidities that are risk factors for the diagnosis and severity of COVID-19 (hospitalization/death due to COVID-19). Overall, its findings highlight that the increased risk of severe symptoms and outcomes for COVID-19 is associated with specific comorbidities and further related to groups of people facing specific psychiatric issues in the UK [27]. Patients with diabetes are associated with higher comorbidity levels from COVID-19 and are significantly at risk for worse disease outcomes and mortality rates too [28].

Regarding the polypharmacy factor and its impact on symptomatology, clinical presentation, the outcome of SARS-CoV-2, and mortality rates, no significant correlation was found in this study, except in the case of the administration of antihypertensive therapy. From the existing literature on polypharmacy, research indicates that polypharmacy is associated with an increased risk of adverse clinical outcomes in patients with COVID-19. Additionally, certain categories of drugs such as antipsychotics, non-tricyclic antidepressants, opioid analgesics, and medications for gastric ulcers and gastroesophageal reflux disease are associated with adverse clinical outcomes in patients with COVID-19 [29].

Our research aligns with previous findings, indicating that patients who succumbed to COVID-19 exhibited significantly higher serum ferritin levels compared to those who survived. Specifically, non-survivors in our study had nearly double the ferritin levels of survivors. This significant elevation in ferritin levels in severe cases may be indicative of hyperferritinemia syndrome, a condition characterized by an overwhelming inflammatory response. Hyperferritinemia has been associated with cytokine storm syndrome, which is a major contributor to mortality in severe COVID-19 cases. Patients with severe COVID-19 should be assessed for signs of hyperinflammation by monitoring laboratory markers such as rising ferritin levels, declining platelet counts, and erythrocyte sedimentation rate trends to help identify patients who may benefit from immunosuppressive treatments, which have the potential to reduce mortality [30].

Ferritin may not merely reflect the inflammatory state but could also play a pathogenic role in the continuation of inflammation. The involvement of ferritin in the iron metabolism and oxidative stress pathways may exacerbate tissue damage, leading to multiorgan failure. Zhou et al. (2020) found that high ferritin levels were associated with increased mortality, suggesting that ferritin is a critical marker of poor prognosis in COVID-19 patients [31]. A study by Bozkurt et al. (2021) confirmed that higher ferritin levels were associated with the need for intensive care and a greater risk of mortality, highlighting their utility in predicting severe outcomes in COVID-19 patients [32].

CRP is another acute-phase protein that has been consistently elevated in patients with severe COVID-19. Our findings show that CRP levels were significantly higher in non-survivors, with values nearly two and a half times greater than those in survivors. This is consistent with studies that have shown elevated CRP levels to be associated with severe disease, including complications such as acute respiratory distress syndrome (ARDS) and myocardial injury, in psychiatric patients [16]. In one study, participants with COVID-19 and depression had greater CRP levels than those with COVID-19 without current major depressive disorder [33]. Additionally, a recent study confirmed that a COVID-19 group of psychiatric patients had higher NLR, ferritin, and CRP levels than those of their control group [34].

The strong correlation between elevated ferritin and CRP levels and poor outcomes in COVID-19 patients suggests that these biomarkers are valuable tools in clinical practice. the Regular monitoring of these levels could assist in identifying patients at higher risk for severe disease progression, allowing for timely and potentially life-saving interventions. For example, patients with significantly elevated ferritin and CRP levels might benefit from the early administration of corticosteroids or other immunomodulatory therapies aimed at curbing an excessive inflammatory response [35].

While this study offers valuable insights into the impact of COVID-19 on psychiatric patients, several limitations should be noted. First, the study was conducted at a single psychiatric facility, which limits the generalizability of the findings to other settings or broader populations. Additionally, the relatively small sample size of 100 patients restricts our ability to draw definitive conclusions, particularly in subgroup analyses based on comorbidities, medication regimens, or vaccination status. Second, the retrospective nature of the study limits its ability to establish causal relationships between variables, such as biomarkers (CRP and ferritin) and patient outcomes. Furthermore, important confounding factors, such as the duration of psychiatric illness, levels of physical activity, or detailed nutritional status, were not available, which could have influenced the results.

Another limitation is the reliance on rapid antigen tests for COVID-19 diagnoses, which may not have been as accurate as PCR tests, potentially leading to false positives or negatives. Additionally, follow-up data on long-term outcomes post COVID-19 recovery were not collected, limiting our analysis of the virus’s potential long-term effects on psychiatric patients.

Given these limitations, future research should aim to include larger, multi-center studies to improve the generalizability of these findings and allow for more robust subgroup analyses. Prospective longitudinal studies would also be beneficial in understanding the long-term impacts of COVID-19 on psychiatric patients, including post-acute sequelae and mental health outcomes. Moreover, future studies should include more detailed data on confounding variables, such as lifestyle factors and comprehensive medical histories, to better clarify the interactions between psychiatric conditions, medications, and immune responses. Additionally, investigating the role of psychotropic medications in modulating immune responses to COVID-19 and other infections in psychiatric populations would be valuable. Finally, research into tailored vaccination strategies and health interventions for psychiatric patients, particularly those with severe mental illness or multiple comorbidities, is crucial to improving health outcomes and reducing the risks posed by pandemics in these vulnerable populations.

## Figures and Tables

**Table 1:** This table provides a comprehensive overview of the demographic characteristics, clinical variables, and outcomes of the study population.

Variable	Category	N	%
Gender	Female	64	64.0%
	Male	36	36.0%
Birthplace	Rural Area	34	34.0%
	Urban Area	66	66.0%
Marital Status	Divorced	9	9.0%
	Married	17	17.0%
	Single	30	30.0%
	Widowed	44	44.0%
Body Mass Index (BMI)	Normal	55	55.0%
	Overweight	12	12.0%
	Obese	27	27.0%
	Morbidly Obese	6	6.0%
Vaccination	1 dose	3	3.0%
	2 doses	74	74.0%
	3 doses	15	15.0%
	Unvaccinated	8	8.0%
Symptoms	Asymptomatic	25	25.0%
	Critical	20	20.0%
	Mild	50	50.0%
	Moderate	3	3.0%
	Severe	2	2.0%
Outcome	Survival	74	74.0%
	Death	26	26.0%
CRP	Normal	71	71.0%
	Abnormal	29	29.0%
Ferritin (Women)	Normal	20	31.3%
	Abnormal	44	68.8%
Ferritin (Men)	Normal	10	27.8%
	Abnormal	26	72.2%
Age	Mean (SD)		75.7 (14.3)

**Table 2:** Chi-square test of the relationship between vaccination and symptoms.

Vaccination Status	Vaccinated	Unvaccinated	*p*-Value
Symptoms			
Asymptomatic/Mild/Moderate	76 (97.4%)	2 (2.6%)	0.001
Severe/Critical	16 (72.7%)	6 (27.3%)	

**Table 3:** Chi-square test of the relationship between vaccination and outcomes.

Vaccination Status	Vaccinated	Unvaccinated	*p*-Value
Outcome			
Survival	73 (98.6%)	1 (1.4%)	<0.001
Death	19 (73.1%)	7 (26.9%)	

**Table 4:** Chi-square tests of the relationship between symptoms and comorbidities.

Comorbidity	Asymptomatic/Mild/Moderate	Severe/Critical	*p*-Value
Hyperuricemia			0.303
-No	75 (78.9%)	20 (21.1%)	
-Yes	3 (60.0%)	2 (40.0%)	
Chronic Obstructive Pulmonary Disease (COPD)			0.032
-No	77 (80.2%)	19 (19.8%)	
-Yes	1 (25.0%)	3 (75.0%)	
Prostatic Hyperplasia			>0.999
-No	74 (77.9%)	21 (22.1%)	
-Yes	4 (80.0%)	1 (20.0%)	
Coronary Artery Disease			0.008
-No	77 (81.1%)	18 (18.9%)	
-Yes	1 (20.0%)	4 (80.0%)	
Renal Failure			0.010
-No	78 (80.4%)	19 (19.6%)	
-Yes	0 (0.0%)	3 (100.0%)	
Heart Failure			0.130
-No	71 (80.7%)	17 (19.3%)	
-Yes	7 (53.8%)	5 (41.7%)	
Thyroiditis			>0.999
-No	72 (78.3%)	20 (21.7%)	
-Yes	6 (75.0%)	2 (25.0%)	
Epilepsy			0.727
-No	67 (77.0%)	20 (23.0%)	
-Yes	11 (84.6%)	2 (15.4%)	
Gastroesophageal Reflux Disease (GERD)			0.220
-No	78 (78.8%)	21 (21.2%)	
-Yes	0 (0.0%)	1 (100.0%)	
Arrhythmias			0.109
-No	60 (82.2%)	13 (17.8%)	
-Yes	18 (66.7%)	9 (33.3%)	
Anemia			0.053
-No	68 (81.9%)	15 (18.1%)	
-Yes	10 (58.8%)	7 (41.2%)	
Osteoporosis			0.220
-No	78 (78.0%)	21 (21.2%)	
-Yes	0 (0.0%)	1 (100.0%)	
Dyslipidemia			0.035
-No	71 (81.6%)	16 (18.4%)	
-Yes	7 (53.8%)	6 (46.2%)	
Diabetes Mellitus			0.310
-No	68 (80.0%)	17 (20.0%)	
-Yes	10 (66.7%)	5 (33.3%)	
Hypertension			0.470
-No	41 (82.0%)	9 (18.0%)	
-Yes	37 (74.0%)	13 (26.0%)	

**Table 5:** Chi-square test of the relationship between symptoms and outcomes.

Outcome	Asymptomatic/Mild/Moderate	Severe/Critical	*p*-Value
Survival	74 (100.0%)	0 (0.0%)	<0.001
Death	4 (15.4%)	22 (84.6%)	

**Table 6:** Chi-square tests of the relationship between outcomes and comorbidities.

Comorbidities	Outcome	Survival	Death	*p*-Value
Arterial Hypertension	No	40 (80.0%)	10 (20.0%)	0.254
	Yes	34 (68.0%)	16 (32.0%)	
Diabetes Mellitus	No	65 (76.5%)	20 (23.5%)	0.207
	Yes	9 (60.0%)	6 (40.0%)	
Dyslipidemia	No	67 (77.0%)	20 (23.0%)	0.094
	Yes	7 (53.8%)	6 (46.2%)	
Osteoporosis	No	74 (74.7%)	25 (25.3%)	0.260
	Yes	0 (0.0%)	1 (100.0%)	
Anemia	No	66 (79.5%)	17 (20.5%)	0.012
	Yes	8 (47.1%)	9 (52.9%)	
Arrhythmias	No	58 (79.5%)	15 (20.5%)	0.070
	Yes	16 (59.3%)	11 (40.7%)	
Gastroesophageal Reflux	No	74 (74.7%)	25 (25.3%)	0.260
	Yes	0 (0.0%)	1 (100.0%)	
Epilepsy	No	63 (72.4%)	24 (27.6%)	0.505
	Yes	11 (84.6%)	2 (15.4%)	
Thyroiditis	No	68 (73.9%)	24 (26.1%)	>0.999
	Yes	6 (75.0%)	2 (25.0%)	
Heart Failure	No	68 (77.3%)	20 (22.7%)	0.073
	Yes	6 (50.0%)	6 (50.0%)	
Renal Failure	No	74 (76.3%)	23 (23.7%)	0.016
	Yes	0 (0.0%)	3 (100.0%)	
Coronary Artery Disease	No	73 (76.8%)	22 (23.2%)	0.016
	Yes	1 (20.0%)	4 (80.0%)	
Prostate Hyperplasia	No	70 (73.7%)	25 (26.3%)	>0.999
	Yes	4 (80.0%)	1 (20.0%)	
Chronic Obstructive Pulmonary Disease (COPD)	No	73 (76.0%)	23 (24.0%)	0.053
	Yes	1 (25.0%)	3 (75.0%)	
Hyperuricemia	No	71 (74.7%)	24 (25.3%)	0.603
	Yes	3 (60.0%)	2 (40.0%)	

**Table 7:** Chi-square test of the relationship between outcomes and medication.

Medication	Outcome	Survival	Death	*p*-Value
Antihypertensives	No	46 (82.1%)	10 (17.9%)	0.042
	Yes	28 (63.6%)	16 (36.4%)	
Antidiabetics	No	65 (76.5%)	20 (23.5%)	0.207
	Yes	9 (60.0%)	6 (40.0%)	
Antithyroid Drugs	No	63 (75.0%)	21 (25.0%)	0.404
	Yes	11 (68.8%)	5 (31.3%)	
Hyperuricemia Treatment	No	72 (75.0%)	24 (25.0%)	0.277
	Yes	2 (50.0%)	2 (50.0%)	
Prostate Treatment	No	74 (74.7%)	25 (25.3%)	0.260
	Yes	0 (0.0%)	1 (100.0%)	
Anemia Treatment	No	59 (75.6%)	19 (24.4%)	0.583
	Yes	15 (68.2%)	7 (31.8%)	
Antidepressants	No	30 (66.7%)	15 (33.3%)	0.170
	Yes	44 (80.0%)	11 (20.0%)	
Antipsychotics	No	27 (65.9%)	14 (34.1%)	0.164
	Yes	47 (79.7%)	12 (20.3%)	
Anxiolytics	No	55 (69.6%)	24 (30.4%)	0.090
	Yes	19 (90.5%)	2 (9.5%)	
Antiepileptics	No	61 (78.2%)	17 (21.8%)	0.098
	Yes	13 (59.1%)	9 (40.9%)	
Antithrombotics	No	59 (76.6%)	18 (23.4%)	0.289
	Yes	15 (65.2%)	8 (34.8%)	
Gastroprotection	No	69 (74.2%)	24 (25.8%)	>0.999
	Yes	5 (71.4%)	2 (28.6%)	
Anti-dementia Drugs	No	57 (73.1%)	21 (26.9%)	0.789
	Yes	17 (77.3%)	5 (22.7%)	
Antiparkinson Drugs	No	74 (74.7%)	25 (25.3%)	0.260
	Yes	0 (0.0%)	1 (100.0%)	
Antiarrhythmics	No	74 (74.7%)	25 (25.3%)	0.260
	Yes	0 (0.0%)	1 (100.0%)	

**Table 8:** *t*-test of the relationship between outcomes and various biomarkers (CRP, BMI, ferritin).

Outcome	Mean CRP (SD)	*p*-Value (CRP)	Mean BMI (SD)	*p*-Value (BMI)	Mean Ferritin (SD)	*p*-Value (Ferritin)
Survival	1.58 (1.96)	0.002	24.6 (4.3)	0.429	246.2 (150.3)	<0.001
Death	3.46 (2.92)		24.8 (4.0)		416.9 (215.9)	

**Table 9:** Log-rank test of the relationship between survival time and clinical characteristics.

Clinical Characteristic	Group	Median Survival Time (Days)	95% Confidence Interval	Log-Rank Test *p*-Value
Vaccination Status	Unvaccinated	18.0	7.9–28.1	0.014
	Vaccinated	35.0	16.0–54.0	
Symptoms	Asymptomatic/Mild/Moderate	50.0	8.4–91.6	<0.001
	Severe/Critical	18.0	11.1–24.9	
Antidepressant Use	No	22.0	17.4–26.6	0.030
	Yes	37.0	11.6–14.3	
Heart Failure	No	28.0	20.6–35.5	0.016
	Yes	18.0	11.9–24.1	
Coronary Artery Disease	No	28.0	13.4–42.6	<0.001
	Yes	12.0	3.4–20.6	
Ferritin Levels—Males	Normal	28.0	-	0.001
	Abnormal	35.0	-	
Ferritin Levels—Females	Normal	18.0	12.0–23.9	0.015
	Abnormal	24.0	0.0–57.7	

## Data Availability

The data used in this study are not publicly available due to privacy and confidentiality concerns. However, the data can be made available upon reasonable request to the corresponding author, provided that appropriate ethical and privacy considerations are addressed.
